# Supplementary material for: Histone Sample Preparation for Bottom-Up Mass Spectrometry: A Roadmap to Informed Decisions
Source: Proteomes. 2021 Apr 21;9(2):17. doi: 10.3390/proteomes9020017 (PMC8167631; doi:10.3390/proteomes9020017)
Supplement: Supplementary file 1 [file proteomes-09-00017-s001.zip › Supplementary data.pdf]

Supplementary data

Supplementary Figure S1. Coverage plots of all core histones from the AQUA heavy histone peptide standard from Li et al [29].

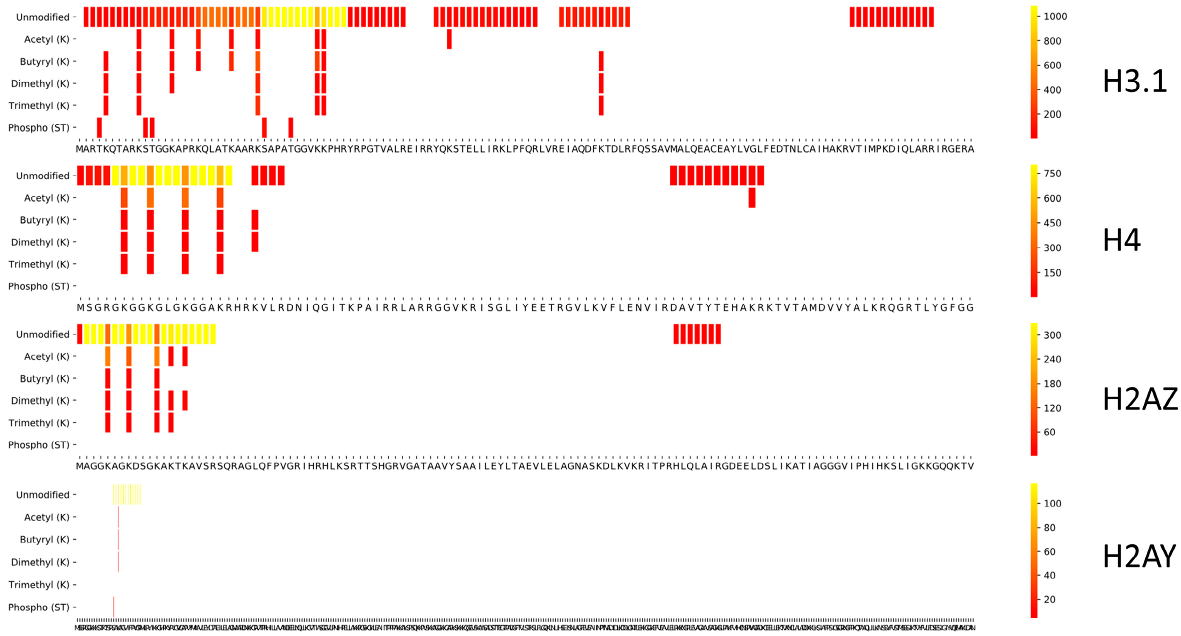

Supplementary Figure S2. Mascot example of peptide H2B 1-29 with multiple possible annotations above scoring threshold for the same mass spectrum. The first hit is reported in the spectrum with a score of 63.4 and expectancy value of 4.6 e-6.

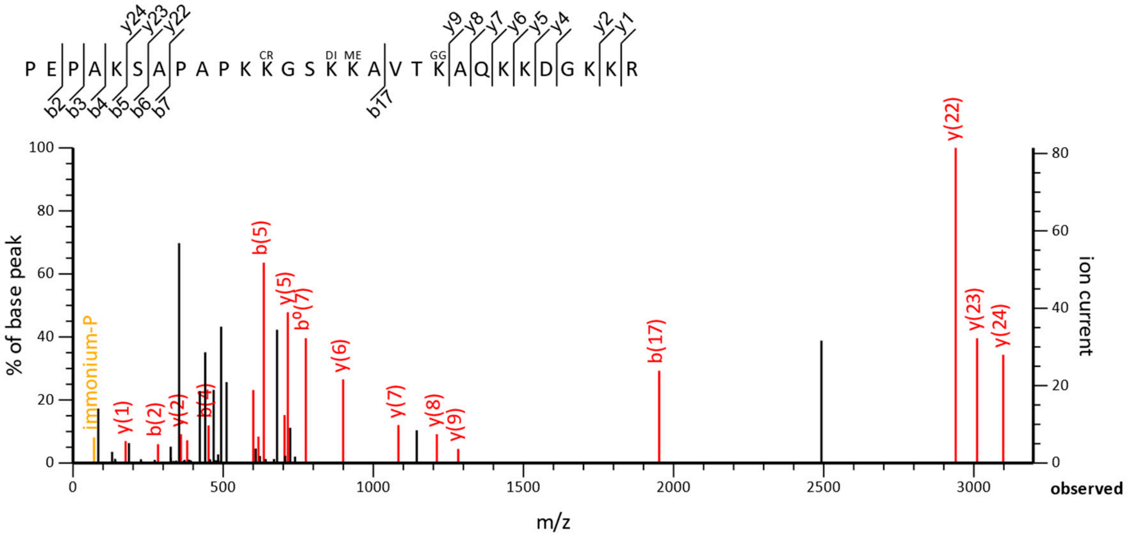

Score	Mr(calc)	Delta	Sequence	Site Analysis
63.4	3731.1350	-0.0253	PEPAKSAPAPKKGSKKAVTKAQKKDGKKR	Methyl KR K16, Crotonyl K12, GG Prop K20, Dimethyl KR K15; 0.02%
63.4	3731.0986	0.0111	PEPAKSAPAPKKGSKKAVTKAQKKDGKKR	
63.4	3731.1350	-0.0253	PEPAKSAPAPKKGSKKAVTKAQKKDGKKR	Methyl KR K11, Crotonyl K12, GG Prop K20, Dimethyl KR K15; 0.02%
60.4	3731.1350	-0.0253	PEPAKSAPAPKKGSKKAVTKAQKKDGKKR	Methyl KR K20, Crotonyl K12, GG Prop K16, Dimethyl KR K15; 0.01%
60.4	3731.0986	0.0111	PEPAKSAPAPKKGSKKAVTKAQKKDGKKR	
60.4	3731.1350	-0.0253	PEPAKSAPAPKKGSKKAVTKAQKKDGKKR	
60.4	3731.1350	-0.0253	PEPAKSAPAPKKGSKKAVTKAQKKDGKKR	
60.4	3731.1602	-0.0505	PEPAKSAPAPKKGSKKAVTKAQKKDGKKR	
60.4	3731.1350	-0.0253	PEPAKSAPAPKKGSKKAVTKAQKKDGKKR	
60.4	3731.1350	-0.0253	PEPAKSAPAPKKGSKKAVTKAQKKDGKKR	Methyl KR K20, Crotonyl K12, GG Prop K11, Dimethyl KR K15; 0.01%

Supplementary Figure S3. Coverage plot of Histone H3.2 for each workflow highlighting the chemical noise and obscured regions introduced by PropTryp.

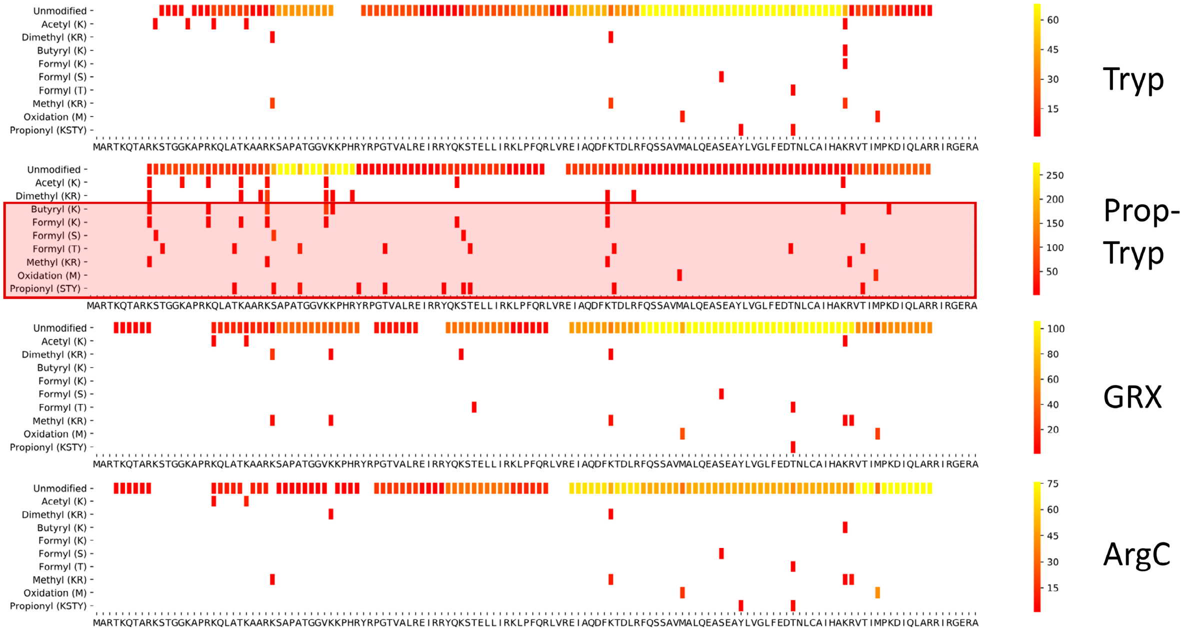

Supplementary Figure S4. Coverage plot of Histone H4 for each workflow highlighting the chemical noise and obscured regions introduced by PropTryp.

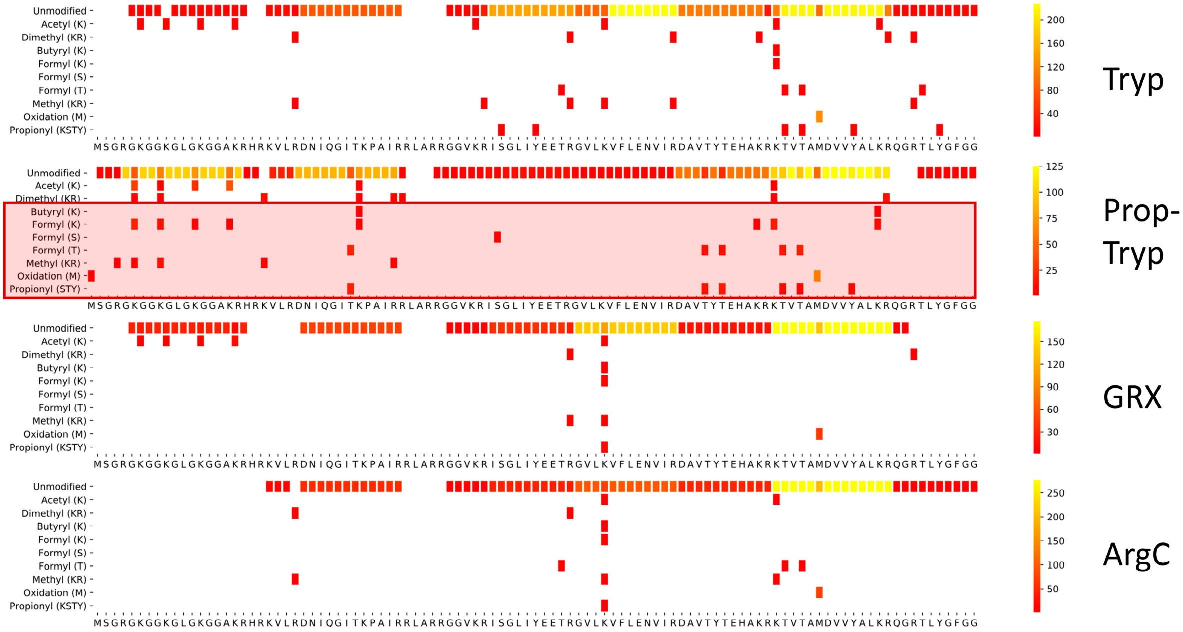

Supplementary Figure S5. Coverage plot of Histone H2A1 for each workflow highlighting the chemical noise and obscured regions introduced by PropTryp.

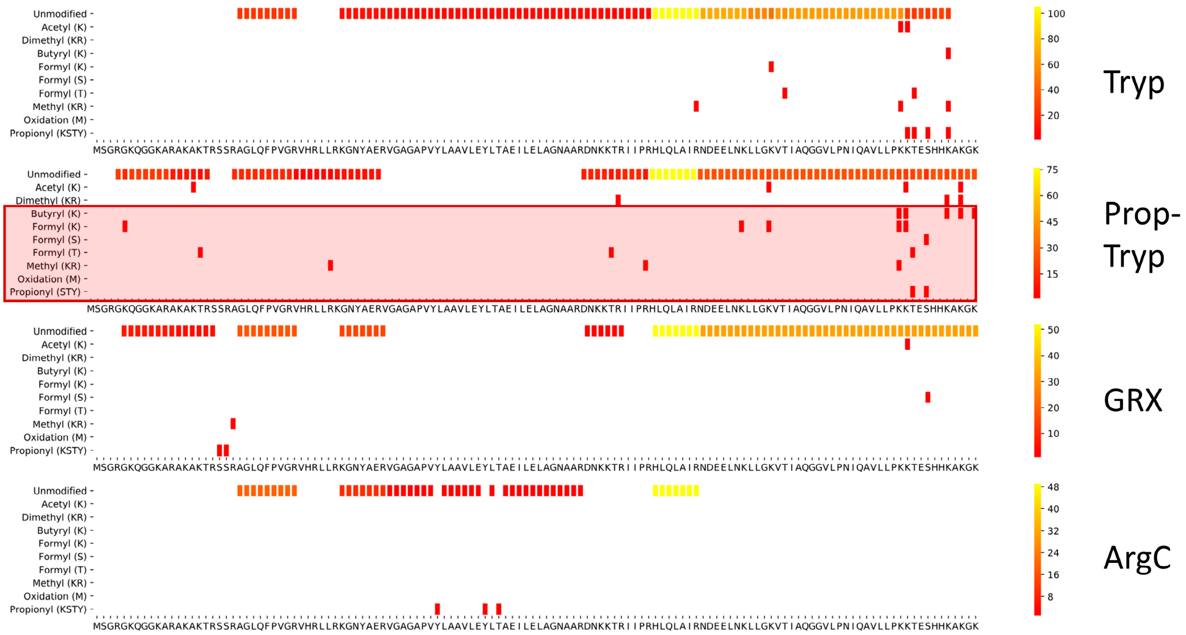

Supplementary Figure S6. Coverage plot of Histone H2B1 for each workflow highlighting the chemical noise and obscured regions introduced by PropTryp.

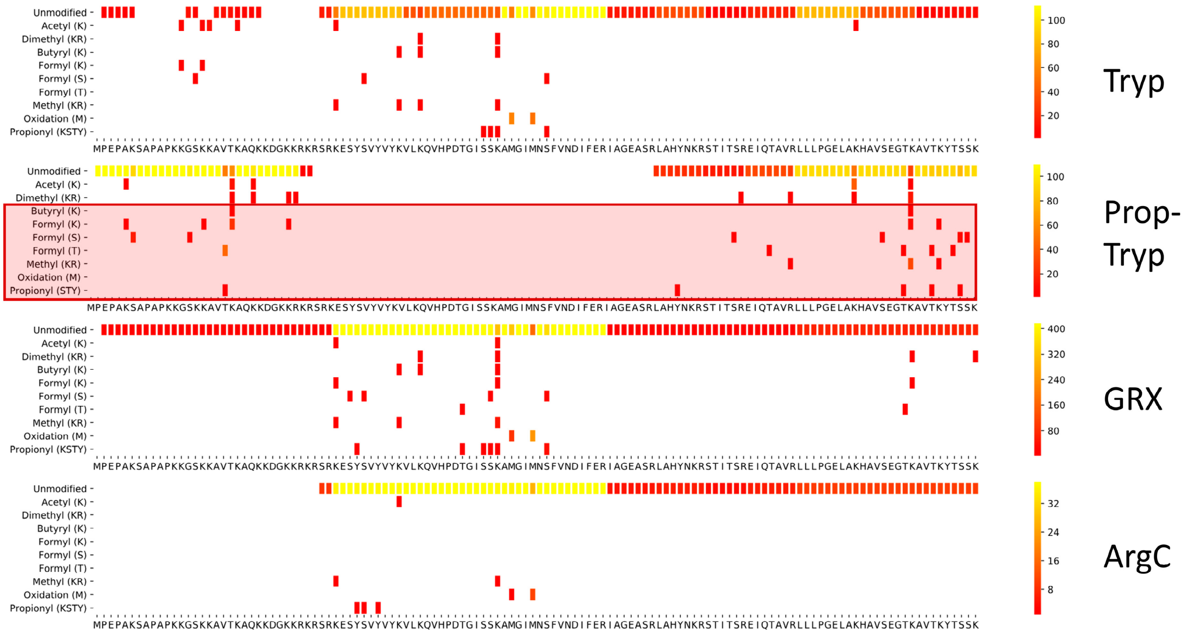

Supplementary Figure S7. Coverage plot of Histone H4 for each workflow with the predicted coverage derived from Uniprot as a reference.

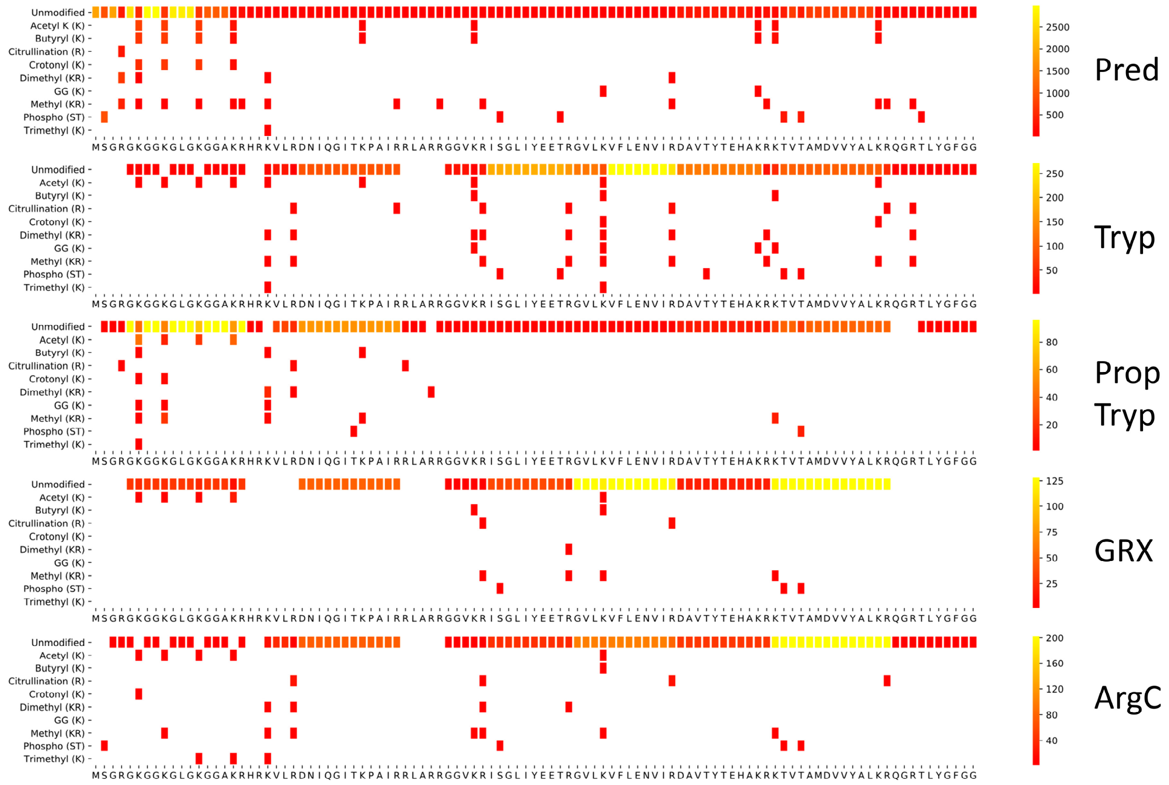

Supplementary Figure S8. Coverage plot of Histone H2A1 for each workflow with the predicted coverage derived from Uniprot as a reference.

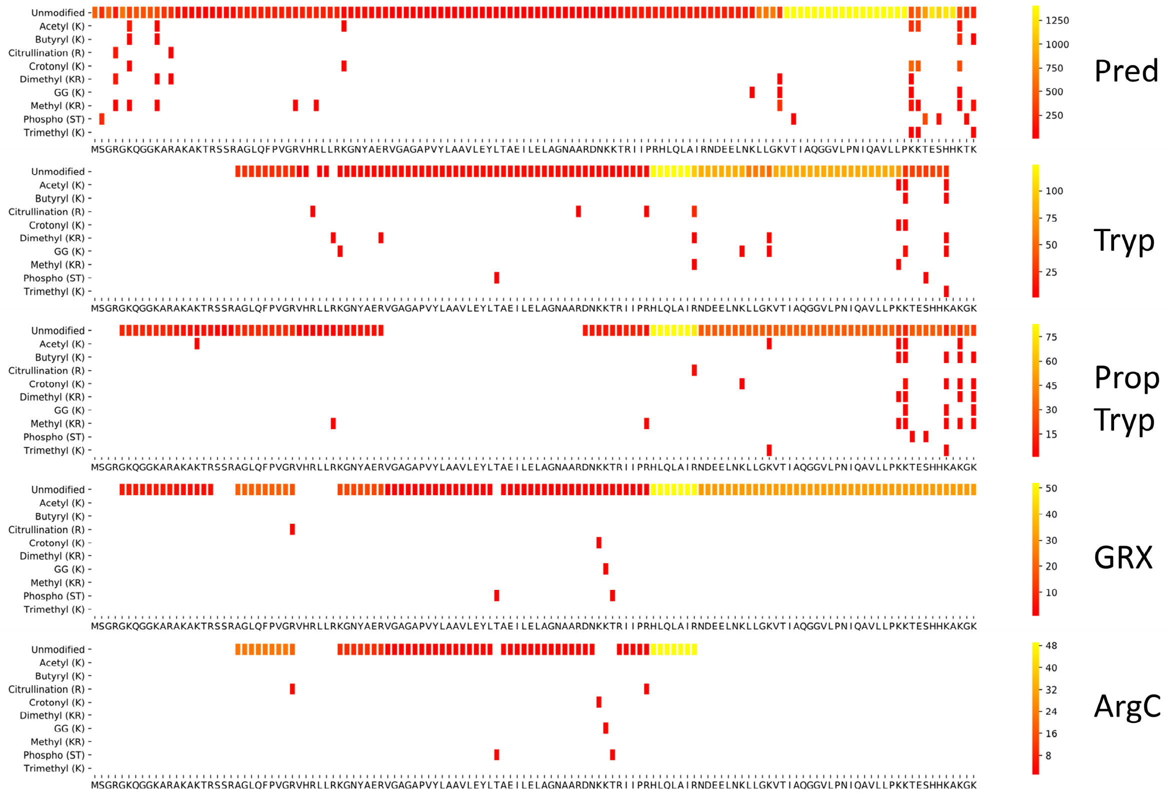

Supplementary Figure S9. Coverage plot of Histone H2B1 for each workflow with the predicted coverage derived from Uniprot as a reference.

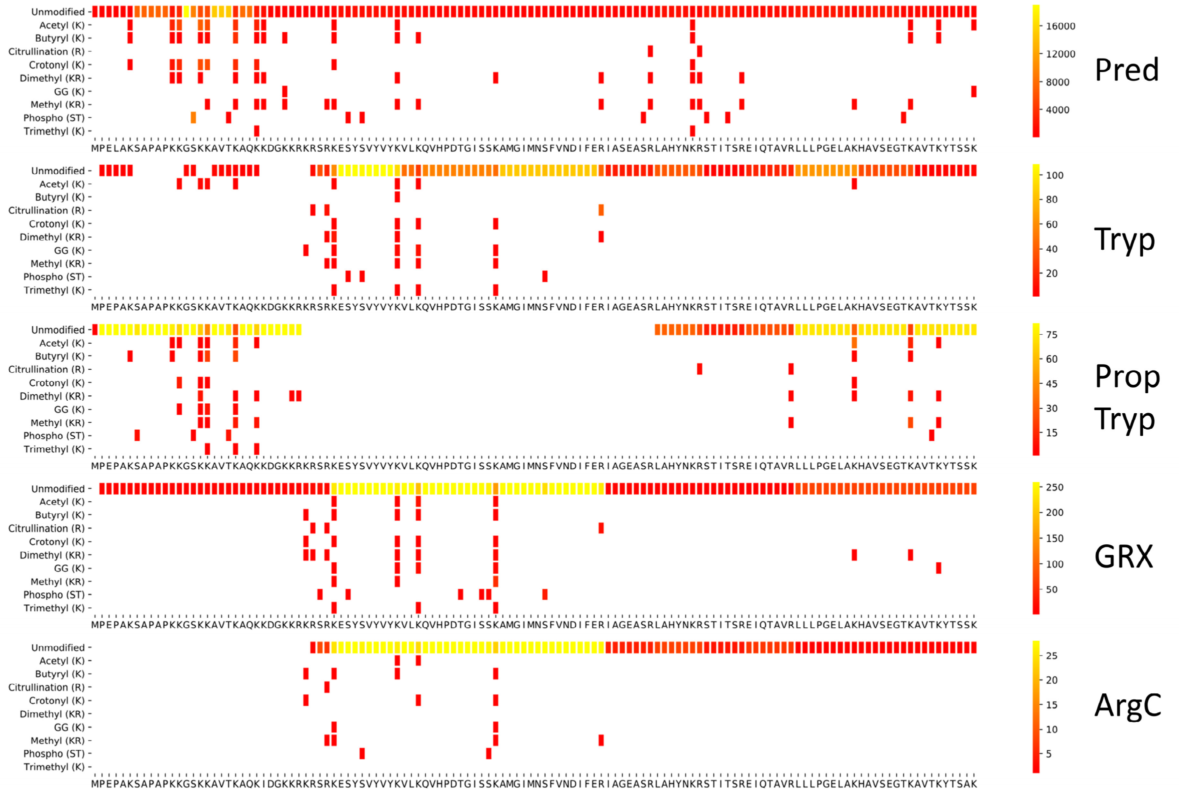

Supplementary Figure S10. Coverage plot of Histone H1.2 for each workflow with the predicted coverage derived from Uniprot as a reference. The ArgC workflow did not cover Histone H1.

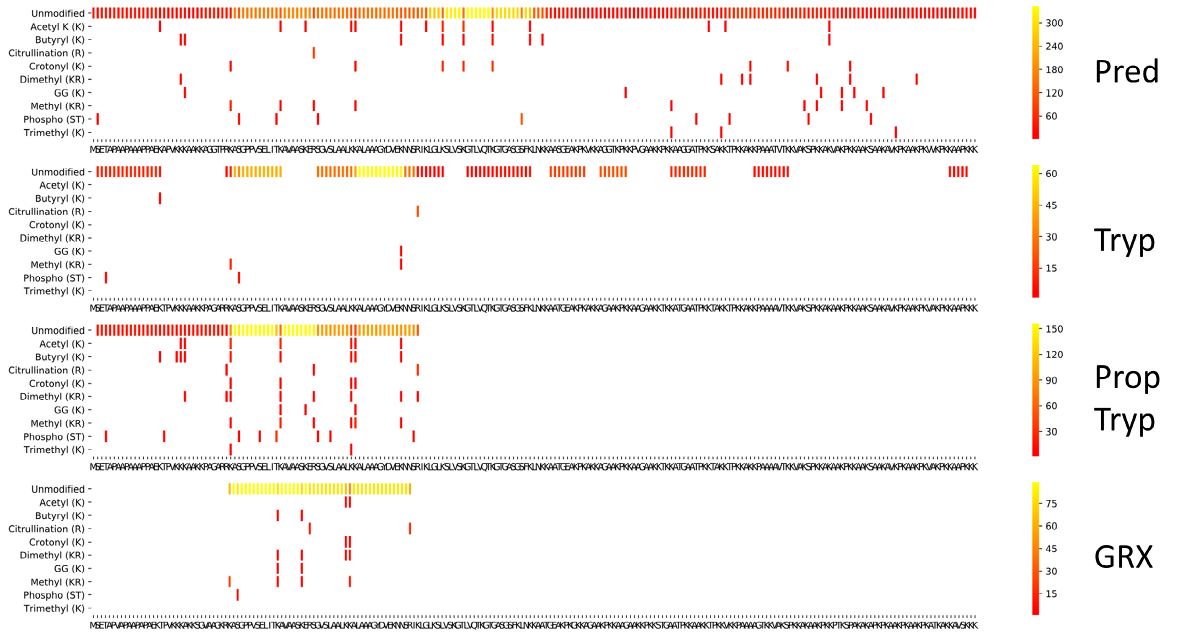

Supplementary Figure S11. Coverage plots of all histone H1 variants detected with the Tryp, PropTryp and GRX workflows.

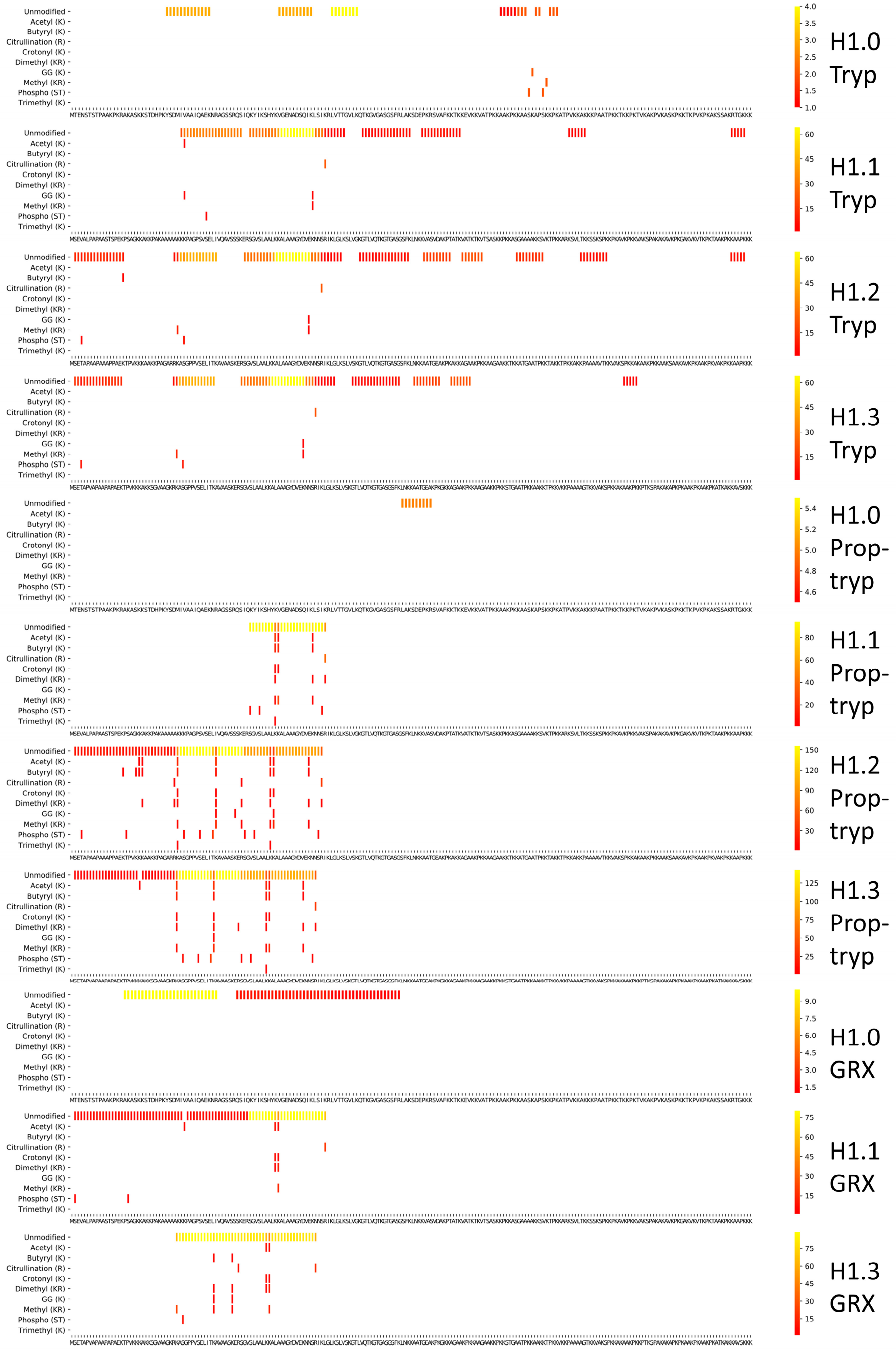

Supplementary Figure S12: feature detectability for different enzymatic treatments. **(A-C)** Two dimensional consensus representations of the different treatments with retention time in the y-axis from top to bottom and the m/z on the X-Axis. Note that the retention time is represented by scan rate and is not linear. Note that the spread of features across the LC gradient is better for PropTryp. **(D)** Charge state distribution of all annotated ions.

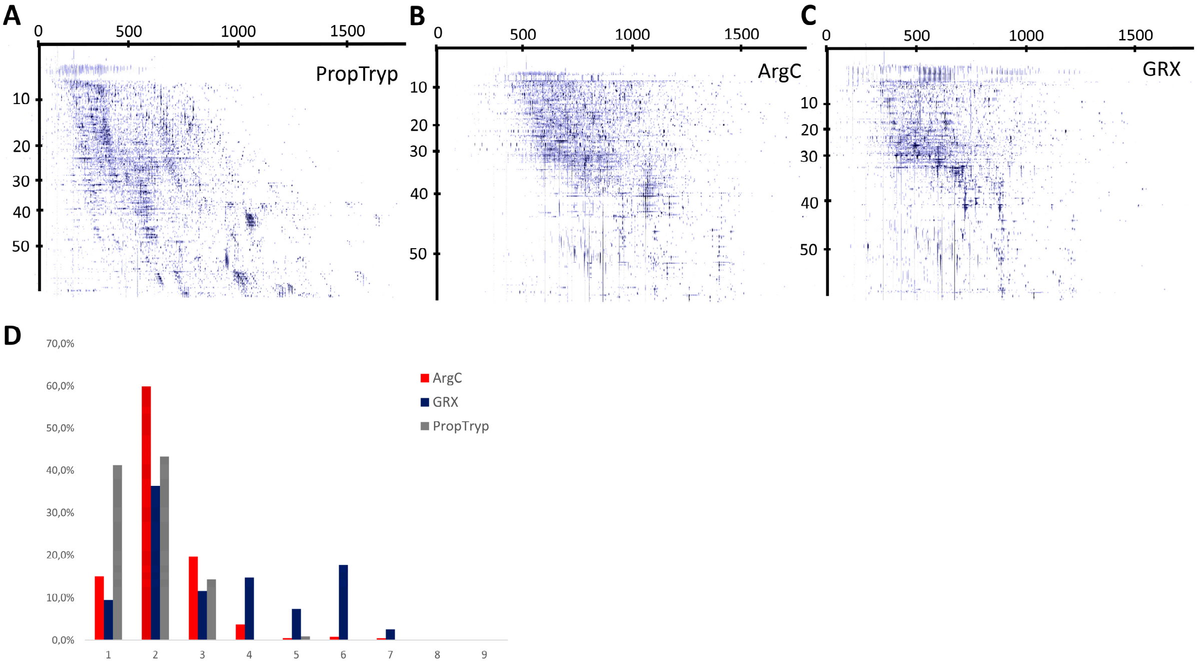

Supplementary Figure S13. Coverage plot of the Semi-specific arginine cleavage search on the ArgC workflow data shows a great coverage of all histone proteins and their modifications. Still, enzymatic aspecificity complicates downstream quantification as shown in Figure 7. The variants displayed are H3.2, H2A1, and H2B1.

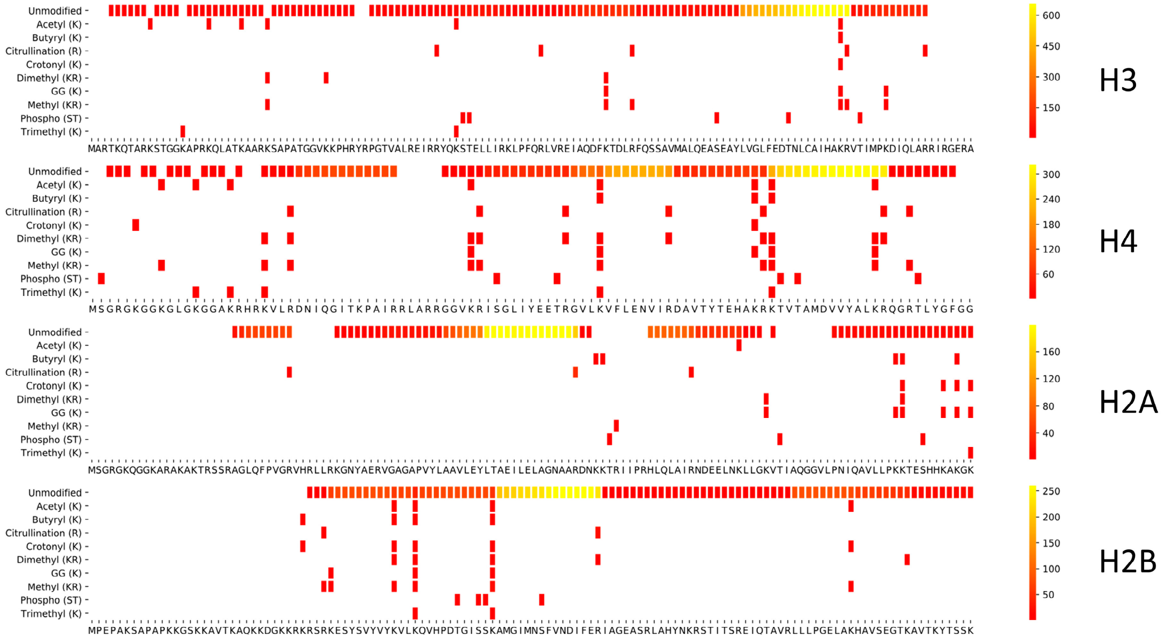

Supplementary Figure S14. Localization of the most prominent peptidoforms of the PropTryp workflow in the 3D ion space of the Progenesis QIP software.

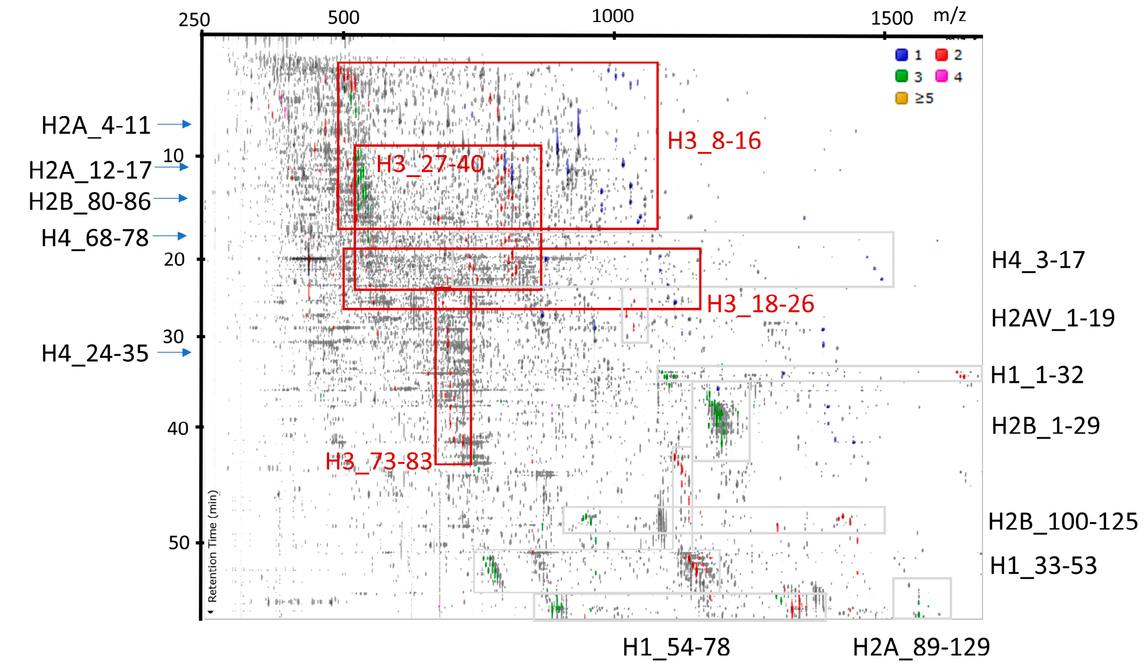

Supplementary Figure S15. Localization of the most prominent peptidoforms of the GRX workflow in the 3D ion space of the Progenesis QIP software.

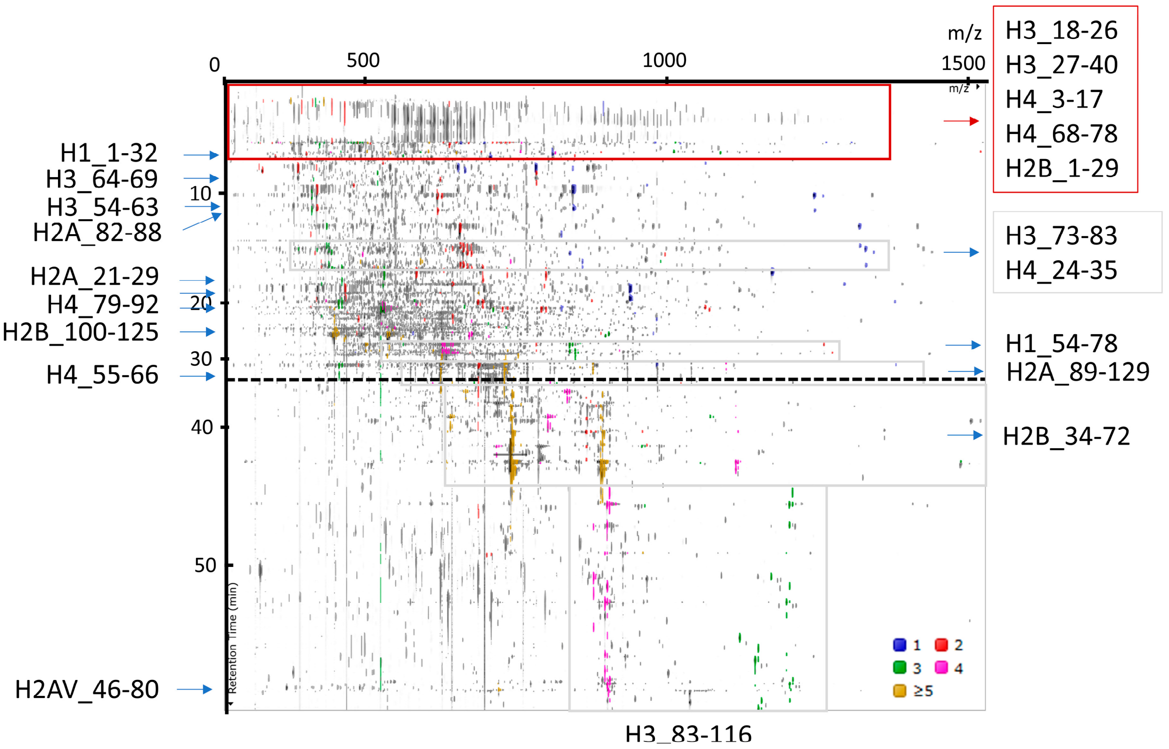

Supplementary table 1. All features exported from Progenesis QIP for all workflows. Detailed legend can be found in the excel file

Supplementary table 2. All annotated features exported from Progenesis QIP for the ArgC, GRX, and PropTryp workflow. Detailed legend can be found in the excel file.
